# Daily image‐guided localization for neuroblastoma

**DOI:** 10.1120/jacmp.v11i4.3388

**Published:** 2010-10-11

**Authors:** Chris Beltran, Atmaram S. Pai Panandiker, Matthew J. Krasin, Thomas E. Merchant

**Affiliations:** ^1^ Department of Radiological Sciences St. Jude Children's Research Hospital Memphis TN USA

**Keywords:** image‐guided localization, cone beam CT, ultrasound, neuroblastoma

## Abstract

The purpose was to quantify the setup margin for pediatric patients with neuroblastoma using cone beam CT imaging (CBCT) and ultrasound localization. Ten patients, with a median age of 4.3 years (1.8 to 7.9) underwent daily pretreatment localization CBCT and every other day post‐treatment CBCT to calculate interfractional and intrafraction movement. Localization was based on CBCT to treatment planning CT registration in the lumbar spine region. Each subject was treated in the supine position under IV general anesthesia using intensity‐modulated radiation therapy. Patients were repositioned based on the daily pretreatment CBCT. Required setup margins based on inter‐ and intrafraction positioning errors were calculated based on weekly and daily imaging scenarios. Four patients had ultrasound localization of the kidneys performed before the CBCT. Correlation between daily CBCT and ultrasound was investigated. A lateral, longitudinal and vertical setup margin of 5.4, 5.6, and 5.9 mm is required without daily CBCT. When daily CBCT was incorporated, the setup margin was reduced to 1.5, 2.1, and 1.7 mm. There was no correlation between the suggested ultrasound shifts and the shifts based on the CBCT. Daily localization based on CBCT of the lumbar spine can reduce the required setup margin for neuroblastoma patients, thereby reducing normal tissue exposure for this young patient population. The internal margin needs further investigation before PTV reduction can be made. Ultrasound localization was highly variable and not correlated to CBCT shifts.

PACS number: 87.53.Jw

## I. INTRODUCTION

Neuroblastoma is an embryonal cancer often presenting as a paraspinal mass associated with the adrenal glands or parasympathetic nervous system. It accounts for 8%–10% of all pediatric tumors and mainly affects children under the age of 5 years.^(^ ^1^ ^)^ Most patients present with metastatic disease and require intensive multimodal therapy that includes surgical resection, high‐dose chemotherapy, and tumor bed irradiation. Unique treatments targeting biological pathways or cell surface proteins have also been developed for neuroblastoma, making it unique amongst pediatric tumors.^(^ ^2^ ^,^ ^3^ ^)^

Total radiation dose in the range of 21 to 26 Gy has been used to address microscopic residual disease after surgery, while guidelines for higher doses have been incorporated into contemporary treatment protocols to address macroscopic residual (ANBL‐0532 Phase III Randomized Trial of Single vs. Tandem Myeloablative as Consolidation Therapy for High‐Risk Neuroblastoma). Recently reported investigations have shown that dose escalation for gross residual may be used to increase local tumor control rates to the 90% level.^(^ ^4^ ^,^ ^5^ ^)^ Comprehensive irradiation of neuroblastoma requires pretreatment knowledge of tumor extent, response to chemotherapy, extent of tumor resection, a detailed understanding of the impact of tumor resection on displacement of critical normal tissue volumes, and normal tissue tolerances of the particular patient.

To limit the dose to normal tissue volumes, radiation therapy for neuroblastoma has evolved over the past two decades from simple anterior‐posterior parallel‐opposed beam arrangements to conformal irradiation of three‐dimensional targets and to highly conformal irradiation using intensity‐modulated radiation therapy (IMRT) methods with limited margins. The most common treatment site is the abdomen and with the typical adrenal or paraspinal location, both kidneys are often associated with the planning target volume (PTV)^(^ ^6^ ^,^ ^7^ ^)^ thus creating a conflict between target coverage and normal tissue avoidance. And while the definition of the gross tumor volume or region at greatest risk for tumor recurrence may be refined with improvements in medical and intra‐operative imaging, reducing the clinical target volume (CTV) and PTV margins currently may have the greatest impact on reducing normal tissue irradiation. The former is more often a protocol‐driven question in a clinical trial, while the latter is based on institutional experience and capability. Understanding the appropriate setup margin, a component of the PTV, is a first and important step as overly large margins may lead to excess dose to normal tissue and toxicity, while inadequate margins may lead to underdosing of the target and treatment failure. The setup margin, which is a component of the PTV along with the internal margin,^(^ ^7^ ^)^ has been studied for different adult abdominal^(^ ^8^ ^–^ ^11^ ^)^ and pelvic^(^ ^12^ ^–^ ^14^ ^)^ sites. There have been no studies that focus on pediatric patients with abdominal tumor location. In this paper we rectify that shortcoming by investigating the proper setup margin for pediatric patients with neuroblastoma. Three localization scenarios were investigated: with and without daily image‐guided localization utilizing cone beam CT (CBCT), and daily localization via ultrasound (US).

## II. MATERIALS AND METHODS

### A. Patient cohort

The first ten neuroblastoma patients enrolled on a prospective IRB‐approved institutional daily localization protocol were included in this study. The median age was 4.3 years (range 1.8 to 7.9 years). Each patient received general anesthesia during treatment, received 23.4 Gy at 1.8 Gy per fraction, and was treated in the supine position. Each patient received IMRT which consisted of, on average, seven equally spaced coplanar fields.

### B. CBCT

The CBCT used for daily localization is a modified version of the Siemens MV‐CBCT (Siemens USA, Concord CA), referred to as the imaging beam line (IBL).^(^ ^15^ ^,^ ^16^ ^)^ This investigational IBL‐CBCT allows for low dose MV‐CBCT to be acquired with sufficient contrast for bony anatomy localization. Under this protocol, the patients received a 1 cGy at isocenter IBL‐CBCT before each treatment (pre‐CBCT) and after every other treatment (post‐CBCT).^(^ ^17^ ^)^ This CBCT has a resolution of 0.36 line pairs per mm and the slice thickness was set to 2 mm. During the simulation CT, patients were immobilized via either a customized vacuum immobilization device or knee cushion (based on the physician's discretion) and visual marks were placed on the patient's body to represent isocenter. At the start of each fraction, the patient was placed on the treatment table and localized via in‐room lasers and the visual marks. Then a CBCT was acquired. This pre‐CBCT was fused to the simulation CT in the Siemens Coherence Adaptive Therapy system, with emphasis placed on the registration of the lumbar spine region nearest the PTV. A mutual information algorithm was used for the initial auto fusion; then the therapist manually adjusted the result if needed. The offset between the pre‐CBCT and the simulation CT were recorded and the patient was shifted appropriately. After every other fraction, a post‐CBCT was acquired and fused in the same manner and the offsets were recorded.

### C. Ultrasound

Given the focus on pediatrics, a nonionizing localization method was investigated, namely ultrasound. Four of the ten patients received a daily localization ultrasound (US) (SonArray, Varian Medical Systems, Palo Alto, CA)^(^ ^18^ ^)^ prior to the pre‐CBCT. The ultrasound system was used to localize the ultrasound image of the patient's kidney to the kidney's position as contoured on the simulation CT. The offsets established by the ultrasound system were recorded. The patients were not localized based on these offset; they were localized based on the pre‐CBCT. After comparison of the US and CBCT on the initial four patients (see Results section below), the US component was discontinued to allow more rapid treatment of this anesthetized population.

### D. Setup margin quantification

Based on the offsets determined in this group of 10 patients, three different localization scenarios were investigated. The first was a simulation of the conventional method of imaging once a week (in this case with CBCT instead of orthogonal port films) and applying a daily adjustment if the offset was greater than 3 mm and a systematic shift if the patient was misaligned by more than 5 mm, per our standard practice. The systematic shift would be the equivalent of remarking the patient for subsequent treatments. The second method, which was the one applied under this protocol, was imaging daily and repositioning the patient if the offset was greater than 2 mm. The third method was based on the offset determined by daily use of the US system. The position determined by the pre‐CBCT was assumed to be the correct setup position. The calculated setup margins for each of these scenarios were composed of two parts. The first component was the interfraction uncertainty, which was based on the pre‐CBCT, and the second component was the intrafraction uncertainty, which was based on the post‐CBCT. The uncertainties were calculated based on the van Herk et al.^(^ ^19^ ^)^ formalism (2.5Σ+0.7σ), where Σ represents the systematic or preparation errors (derived from the standard deviation of the mean offsets) and σ represents the execution or random error (derived from the root mean square of standard deviations of the offsets). The two components were combined (Eq. (1)) to give the full setup margin:

(1) $$2.5\sqrt{\Sigma_{inter}^{2}+\Sigma_{intra}^{2}}+0.7\sqrt{\sigma_{inter}^{2}+\sigma_{intra}^{2}}$$

For the daily CBCT imaging scenario, the interfraction motion was assumed to be 0, so only the intrafraction component contributes to the setup margin. For the other two scenarios, both the interfraction and intrafraction components are taken into account.

## III. RESULTS

For each patient, there were 13 pretreatment CBCTs and seven post‐treatment CBCTs. The median CTV was 125.2 cc (range 84.1 to 251.1 cc). The interfraction component of the setup margin based on once a week imaging with a 5 mm threshold for a systematic shift was 5.2 mm lateral (right‐left), 5.2 mm longitudinal (ant‐post), and 5.6 mm vertical (sup‐inf). The intrafraction component of the setup margin was 1.5 mm lateral, 2.1 mm longitudinal, and 1.7 mm vertical. When this intrafraction motion is incorporated, the full setup margin for once a week imaging becomes 5.4 mm lateral, 5.6 mm longitudinal, and 5.9 mm vertical.

**Figure 1:**
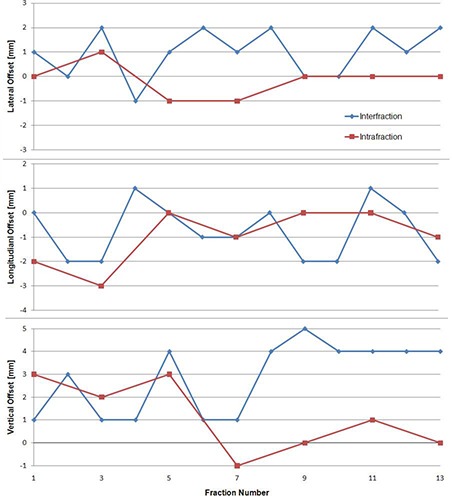
Plot of daily inter‐ and intrafraction setup error for a typical neuroblastoma patient.

With daily image‐guided localization, the interfraction setup margin was considered to be 0, and only the intrafraction motion contributes to the setup margin. The intrafraction component of the setup margin was 1.5 mm lateral, 2.1 mm longitudinal, and 1.7 mm vertical. Table 1 gives a breakdown of the individual components of the margin calculation. Figure 1 is a plot of the daily inter‐ and intrafraction setup error in each direction for a typical neuroblastoma patient. There was no statistical difference between the two immobilization techniques.

**Table 1:** Orthogonal components used for the calculation of a setup margin based on the data from ten patients.

	*Interfraction*			*Intrafraction*			*Combined*		
*(mm)*	*Lat*	*Long*	*Vert*	*Lat*	*Long*	*Vert*	*Lat*	*Long*	*Vert*
Mean	0.1	‐0.2	0.5	0.0	0.4	0.5	‐	‐	‐
Σ	1.5	1.4	1.7	0.4	0.6	0.4	1.5	1.5	1.7
σ	2.2	2.4	2.1	0.8	1.0	1.0	2.3	2.6	2.3
Margin	5.2	5.2	5.6	1.5	2.1	1.7	5.4	5.6	5.9

Interfractional: motion between daily radiation treatments; Intrafractional: motion within a daily radiation treatment; Combined: the combined inter‐ and intrafractional motion.

Σ represents the systematic or preparation errors and σ represents the execution or random error.

US localization was obtained on the first four patients in conjunction with CBCT. Based on the high variability of daily shifts and lack of correlation with the CBCT, US was discontinued in this population as a method of localization and only CBCT was continued on this study.

**Figure 2:**
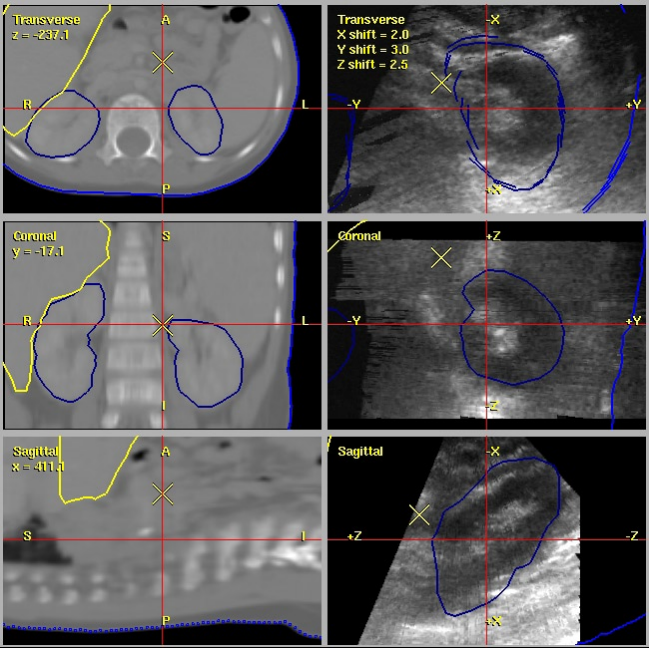
An image of the US to CT kidney registration for a neuroblastoma patient. The left side of the the simulation CT with the contour of the kidneys and liver shown. The right side is the US image of the kidney with the simulation CT kidney contour shown. Each image represents the isocenter slice with isocenter represented by the large X.

**Figure 3:**
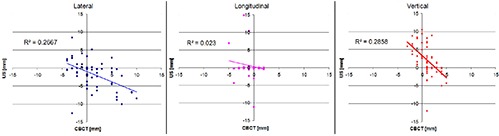
A scatter plot of the CBCT vs. US recommended shifts for each direction: lateral (right‐left), longitudinal (ant‐post), and vertical (sup‐inf). As can be seen with the aid of the Spearman correlation coefficient $\left(R^{2}\right)$, there is no correlation between the CBCT and US recommended shifts.

The interfraction setup margin based on US for these four patients was 8.4 mm lateral, 4.8 mm longitudinal, and 6.8 mm vertical. Figure 2 is an example image of the US to CT registration. The left side of the figure is the simulation CT with the contour of the kidneys and liver shown. The right side is the US image of the kidney with the simulation CT kidney contour shown. Each image is on the isocenter slice; isocenter is represented by the large X in the images. Scatter plots of the suggested US shifts vs. the CBCT shifts for each direction are shown in Fig. 3, along with the Spearman coefficient of determination value $\left(R^{2}\right)$.

## IV. DISCUSSION

Daily image‐guided localization can help reduce the setup margin required for neuroblastoma patients and is therefore recommended. Using a conservative estimate, a 6 mm symmetric setup margin would be required if conventional weekly imaging is used. If effective daily image‐guided localization is used, the setup margin can be safely reduced to 2 mm. This reduction is crucial because highly conformal treatments, such as IMRT, are routinely being used to treat these patients and as conformality increases so does the possibility of a dosimetric target miss without proper margins. It is important to note that this is not a PTV reduction, as the internal margin is not included in this calculation. The ability to image with low dose is crucial for pediatric patients. Daily imaging with the IBL‐CBCT system delivers approximately the same dose to the patient as traditional weekly orthogonal portal images.^(^ ^15^ ^)^ The combination of reduced margins and highly conformal treatments is very desirable. The median size of the neuroblastoma CTV for the patients in this study was 125 cc. Assuming spherical geometry, a setup margin expansion of 6 mm would increase the target volume to about 212 cc, a 70% increase. With a 2 mm expansion, the volume only increases to approximately 150 cc, a 20% increase. This corresponds to sparing over 60 cc of normal tissue that would otherwise be targeted. Although this study focused on neuroblastoma, the general results may be applicable to other diseases which occur in the abdomen area given a similar patient population, namely pediatric patients treated in the supine position and anesthetized.

There exists concern about both tumor bed and kidney motions due to breathing. The inclusion of an individual assessed internal margin^(^ ^7^ ^)^ based on 4D CT scans have recently been introduced into our clinic. These scans are currently being analyzed for a possible population suggested internal margin. Preliminary data show that kidney motion for pediatric patients is less than that for adults; approximately 1 mm ant‐post and 2–3 mm sup‐inf.^(^ ^20^ ^)^ This is around the same size as the setup margin using daily CBCT localization; therefore, it is crucial that the internal margin be incorporated into the PTV if the smaller 2 mm setup margin is used. A new treatment protocol is being introduced that will call for implantation of gold fiducials during resection that will mark the extent of the tumor bed. In addition to aiding in the contouring of the target, these fiducials will also be used for daily localization. This will serve to address another concern, namely that the lumbar spine may not be an ideal surrogate for target location.

Ultrasound proved ineffective as a localization method for these patients. This is contrary to Fuller et al.^(^ ^21^ ^)^ who found US localization acceptable for gallbladder carcinoma. However, this is similar to results found in prostate therapy.^(^ ^14^ ^–^ ^22^ ^,^ ^23^ ^)^ There was hope that since the kidney is somewhat more superficial than the prostate (especially for pediatric patients), that the system would be effective. It is conceivable that if the US system were used more frequently and the therapist had additional training, better results may have been obtained. Also, the US may have captured the kidney at a phase in the breathing cycle different than baseline, which may have added to the large discrepancy with CBCT. If this is the case, however, it implies that the localization to the kidney may not lead to the mean position that the treatment plan is based on. Given that we treat at most one neuroblastoma patient per month, we had poor results based on the four patients we quantitatively evaluated, and based on the poor results reported with prostate patients, we decided to discontinue the use of the US system.

## V. CONCLUSIONS

Daily localization based on CBCT of the lumbar spine can effectively reduce the required setup margin from 6 to 2 mm for neuroblastoma patients compared to conventional weekly imaging, and it is therefore recommended. Full quantification of the internal margin is needed before PTV margin reduction is introduced. Ultrasound of the kidneys was ineffective as a method of localization for this patient population.

## ACKNOWLEDGEMENTS

This research was partially funded by a grant from Siemens Medical Systems and by support from the American Lebanese Syrian Associated Charities (ALSAC).
